# EEG Quantification: A New Dawn for an Old Tool

**DOI:** 10.31083/RN52778

**Published:** 2026-06-29

**Authors:** Jesús Pastor

**Affiliations:** ^1^Clinical Neurophysiology, Hospital Universitario La Princesa, 28006 Madrid, Spain; ^2^Biomedical Research Institute, Hospital Universitario La Princesa, 28006 Madrid, Spain

The human brain has evolved to manage information through the modulation of electrical activity. Given that the physiological function of this organ underlies emotions, reasoning, perception, behavior, and consciousness, and that pathological brain functioning causes neurological and psychiatric diseases, the objective assessment of brain (mal) functioning has considerable medical and scientific value [1].

The dynamics of brain activity reflect interactions between different (and usually anatomically segregated) neural networks [2]. Electroencephalography (EEG)—invented one century ago by the German psychiatrist Hans Berger—is the fastest, least expensive, and most portable method for noninvasive brain activity assessment and one of the oldest ancillary diagnostic methods currently used in medicine [3,4]. Since its discovery, EEG has gained widespread use, with almost every hospital of sufficient size and/or complexity now having an electroencephalograph. EEG recordings are visually analyzed by clinical neurophysiologists in a way that has barely changed since the invention of this technique. This form of analysis has influenced the development of EEG, making it a tool primarily oriented toward the study of epilepsy. Among the PubMed papers on EEG, more than 50% are related to epilepsy, and less than 12% are related to psychiatric and dementia disorders [5]. This disparity reflects the fact that identifying high-amplitude sharp waves—hallmarks of epilepsy—is easier than assessing low-amplitude focal slowing in subcortical lesions or subtle changes in patients with dementia. These subtle changes, many of which are related to modifications in synchronization, cannot be assessed even by highly experienced neurophysiologists.

In addition to being largely restricted to epilepsy, EEG is sometimes viewed as a subjective method, with analysis outcomes strongly depending on the interpreter [6,7,8]. Attempts [9] have been made to reduce inter-rater variability by introducing a consensus for EEG interpretation and thus making EEG analysis more objective; however, the involved variables are mostly qualitative or binary (present/absent), and specificity is therefore not very high.

In recent decades, neuroscientists, clinicians, mathematicians, physicists, and engineers (among other experts) have sought ways of overcoming these limitations to extend the application scope of EEG beyond epilepsy and reduce diagnostic subjectivity. One of the main options is the development of quantitative EEG (qEEG), in which case mathematical tools are used to increase the reliability of and expand the information extracted from neurophysiological recordings [5]. This approach refers to the use of any type of post hoc numerical method to obtain results pertaining to the frequency spectrum, synchronization, network dynamics, or anything else from a bioelectrical magnitude and has rapidly gained popularity (see Chapter 13 in ref. [5]). However, the use of qEEG in clinical practice is not frequent, except for intensive care units (ICUs), in which case long-term monitoring continuous EEG (cEEG) and qEEG are gaining popularity [4,10,11,12].

**Fig. 1.**
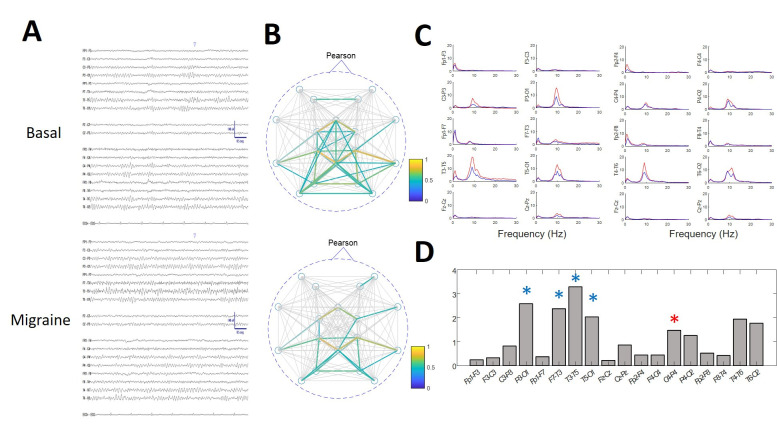
**qEEG findings obtained during a migraine attack**. (A) Raw recordings obtained in the basal state (top) and during a migraine attack (bottom). (B) Connectograms displaying Pearson’s correlation coefficients for recordings obtained in the basal state (top) and during a migraine attack (bottom). (C) Average spectra obtained in the basal state (blue) and during a migraine attack (red) for all channels. (D) Area difference between basal- and migraine-state spectra for different channels. qEEG, quantitative electroencephalography.

In clinical practice, the initial diagnosis based on resting-state electroencephalography (rsEEG) is commonly modified after qEEG analysis, as this analysis helps identify changes not observable in raw recordings. Fig. 1 shows an important example of the effectiveness of qEEG in a scenario where EEG recordings are obtained under basal conditions and during a migraine attack. In both cases, rsEEG results reveal physiological features (Fig. 1A). Connectograms indicate a notable decrease in the number of links during the migraine attack (Fig. 1B) and a concomitant considerable increase in alpha power in the left parieto-temporo-occipital junction (Fig. 1C). A comparison of symmetrical channel pairs reveals a marked increase in absolute area for power spectra during the migraine in left parieto-occipital (P3-O1), frontotemporal (F7-T3), temporal (T3-T5), and temporo-occipital (T5-O1) channels (blue asterisks) and an increase in the right centroparietal (C4-P4) (red asterisk).

The above example illustrates the superiority of qEEG over rsEEG. However, migraine attacks are not the only type of pathology for which qEEG provides valuable insights, with further examples provided below [5].

*Epilepsy*

EEG and its variants, such as video-EEG, cEEG, or electrocorticography, are most commonly used. Various numerical tools are employed to identify epileptic sources (e.g., low resolution electromagnetic tomography) or identify and quantify epileptic seizures/status epilepticus (ES/SE) in ICU patients [13]. However, qEEG can do much more than this. Interictal recording analysis helps identify alterations in spectral composition or synchronization that can facilitate diagnosis, even in the absence of epileptogenic activity. These background alterations persist during sleep, indicating an established alteration in brain dynamics. qEEG makes the diagnosis of ES/SE more reliable, especially when subtle changes are present [5].

*Critical Patients*

ICU patients are subjected to sedation and analgesia and often have primary brain involvement and/or severe ionic or metabolic abnormalities, thus having severely altered brain dynamics. This impairment of brain physiology complicates visual EEG-based diagnosis. Numerical analysis can reveal the existence of these changes, causing the modification of the initial diagnosis and the functional prognosis of the patient. Furthermore, neuromuscular blockade increases recording quality and thus facilitates analysis. Finally, the use of quantitative approaches in cEEG not only provides valuable information pertaining to ES/SE but is also very important for the analysis of other pathologies (e.g., the evolution of encephalopathies and focal ischemia) and treatment titration [14].

*Encephalopathies*

In general, rsEEG in encephalopathies presents nonspecific characteristics. Visual EEG changes are usually imperceptible because the clinical course is typically slow. However, qEEG can detect subtle changes, thus helping refine prognosis before clinical modifications are observed [15].

*Dementias and Psychiatric Pathologies*

The utility of conventional rsEEG for diagnosing psychiatric disorders or differentiating between specific types of dementia is debated. Nonetheless, objective qEEG measurements offer potential improvements in these fields. Numerous changes in spectral composition and synchronization have been observed for these types of pathologies, which encourages further investigation into the numerical characterization of psychiatric pathologies and dementias [16].

qEEG is expected not only to facilitate ES/SE monitoring in ICUs, its main current application, but also to expand EEG use to other pathologies, improving diagnostic capacity and specificity and potentially transforming its clinical role. However, an in-depth study of the consistency and standardization of different qEEG tools is necessary. Given the need to perform numerous studies on different pathologies, multicenter collaboration is required to ensure diagnostic objectivity and accuracy.

Several aspects, such as the use of different numerical analysis methods, the degree of interchangeability between them, and the levels of standardization among centers, still need clarification. Fortunately, these unresolved problems are being addressed through standardization efforts [9].
